# The data of TLR8 species specific downstream differentially regulated genes (DEGs)

**DOI:** 10.1016/j.dib.2019.104314

**Published:** 2019-08-02

**Authors:** Da Ao, Jianzhong Zhu

**Affiliations:** a Comparative Medicine Research Institute, Yangzhou University, China; b College Veterinary Medicine, Yangzhou University, China; c Jiangsu Co-innovation Center for Prevention and Control of Important Animal Infectious Diseases and Zoonoses, China; d Joint International Research Laboratory of Agriculture and Agri-Product Safety, Yangzhou, 225009, China

**Keywords:** Toll-like receptor 8, R848, Porcine, DEGs

## Abstract

To compare porcine TLR8 (pTLR8) and human TLR8 (hTLR8) signaling pathways and downstream genes, we activated the pTLR8 and hTLR8 reporter cells with TLR8 agonist R848, and subjected the stimulated cells together with non-stimulated control cells for transcriptome analysis. There are 1157 differentially expression genes (DEGs) in R848 activated hTLR8 cells, whereas 502 DEGs in R848 activated pTLR8 cells. Among these DEGs, 804 genes are hTLR8 specific, 149 genes are pTLR8 specific, and 353 genes are hTLR8 and pTLR8 common. Related Results were published in reference [Ao, 2019].

**Table:** Specifications Table

Subject area	*Biology*
More specific subject area	*Toll-like receptor signaling*
Type of data	*Tables*
How data was acquired	*Transcriptome analysis*
Data format	*Filtered*
Parameters for data collection	*Corrected P-value less than 0.05 and absolute fold change of 2 were set as the threshold for significantly differential expression.*
Description of data collection	*A total amount of 3 μg RNA per sample was used as input material for the RNA sample preparations. Sequencing libraries were generated using NEBNext® UltraTM RNA Library Prep Kit for Illumina® (NEB, USA). After cluster generation, the library preparations were sequenced on an Illumina Hiseq platform and 125 bp/150 bp paired-end reads were generated. Clean reads were obtained by removing reads containing adapter, reads containing poly-N and low-quality reads from raw data. Paired-end clean reads were aligned to the reference genome using Hisat2 v2.0.5. The read number mapped to each gene was counted using FeatureCounts v1.5.0-p3, and then FPKM (Fragment Per Kilobase of transcript sequence per Millions base pairs sequenced) of each gene was calculated based on the length of the gene and read count mapped to this gene, which is used to estimate gene expression level. Differential expression analysis of two conditions was performed using the edgeR R package.*
Data source location	*Yangzhou, Jiangsu, China*
Data accessibility	*with the article*
Related research article	Ao D, Xia P, Jiang S, Chen N, Meurens F, Zhu J. Comparative transcriptome analysis of TLR8 signaling cells revealed the porcine TLR8 specific differentially expressed genes.*Dev Comp Immunol.* 2019 Sep; 98:129–136. https://doi.org/10.1016/j.dci.2019.05.004.

**Table:** 

**Value of the data** • The data contain information of both human and porcine TLR8 specific downstream genes, reflecting the species specificity and potential new functions of TLR8 signaling. • Our data will benefit those who work on comparative immunology and innate immunology. • Among the information of DEGs we generated, some downstream DEGs can be directly linked with human TLR8 and/or porcine TLR8, whereas other DEGs can feed-back modulate human TLR8 and/or porcine TLR8 signaling. • In addition to protein coding genes in DEGs, there are DEGs for non-coding RNA such as lincRNA, miRNA and anti-sense RNA etc., which suggests cross-talks between TLR8 signaling and these different types of non-coding RNAs.

## 1 Data

The human TLR8 specific DEGs were shown in Supplementary Table 1. Totally, there are 804 hTLR8 specific DEGs, among which some DEGs are upregulated, whereas others are downregulated. The upregulations of RNF169, IRS4, SHPRH, and SRXN1 were confirmed by qRT-PCR in the hTLR8 reporter cells but not in porcine source alveolar macrophages (PAMs). These confirmed upregulated DEGs were marked in red colors in Supplementary Table 1.

The porcine and human TLR8 common DEGs were shown in Supplementary Table 2. Four from total 353 DEGs were picked and confirmed to be upregulated by qRT-PCR in both reporter cells, THP-1 and PAMs. These four upregulated DEGs are MATR3, IGIP, SAT1, YBX2, respectively, which were marked in red colors in Supplementary Table 2.

The 149 porcine TLR8 specific DEGs were shown in Supplementary Table 3. The upregulations of pTLR8 specific DEGs HIST2H4B, PLPP2, DDX47 and VAMP5 were confirmed by qRT-PCR in pTLR8 reporter cells and PAMs, but not in human source THP-1 cells. In addition, the GO enriched DEGs contain 7 DEGs; among these, CATSPERG, HAVCR2, CNTN2 were upregulated and confirmed by qRT-PCR in pTLR8 reporter cells and PAMs, but not in THP-1 cells. The two categories of DEGs were marked with red and purple colors, respectively in Supplementary Table 3.

## 2 Experimental design, materials and methods

### 2.1 Development of human and porcine TLR8-NF-κB dual-luciferase reporter cells

We established the TLR8-NF-κB dual-luciferase reporter cells using the following method [1]. Specifically, HEK293 cells in 6-well cell culture plate (0.5 × 10^6^/well) were infected at multiplicity of infection (M.O.I.) of 1 with lentivirus expressing NF-κB firefly luciferase (Fluc) (G&P biosciences, Santa Clara, CA, USA). Forty-eight hours post infection (p.i.), the infected cells were selected with 10 μg/ml Blasticidin (InvivoGen). The individual cell clones from the selected cells were characterized using the TNF-α stimulation. The picked highest responsive cell clone was used for co-transfection with plasmids TK Renilla luciferase (Rluc) (Promega) and pBabe hygro (Addgene) using the ration of 20:1. The transfected cells were selected with 100 μg/ml hygromycin (InvivoGen) and the selected cell clones were characterized by the stimulation of TNF-α, R848, IL-1β and PMA to obtain the NF-κB Fluc/Rluc dual-luciferase reporter cells with specific response to TNF-α, IL-1β and PMA but not to R848. The above obtained NF-κB Fluc/Rluc reporter cells were transfected with pcDNA3.1 porcine TLR8 (pTLR8) and human TLR8 (hTLR8), respectively, and then the transfected cells were selected with 800 μg/ml G418 (InvivoGen). The individual cell clones from the G418 selection were characterized by stimulation of R837, R848, PMA and other TLR and NOD agonists to obtain TLR8 specific dual-luciferase reporter cells.

### 2.2 Transcriptome analysis

The pTLR8 and hTLR8 dual-luciferase NF-κB reporter cells were stimulated for 12h with TLR8 agonist, R848 respectively. The stimulated cells together with non-stimulated control cells were subjected for transcriptome analysis. Transcriptome analysis was performed in Novogene (Beijing, China) as following [2]. A total amount of 3 μg RNA per sample was used as input material for the RNA sample preparations. Sequencing libraries were generated using NEBNext® UltraTM RNA Library Prep Kit for Illumina® (NEB, USA). After cluster generation, the library preparations were sequenced on an Illumina Hiseq platform and 125 bp/150 bp paired-end reads were generated. Clean reads were obtained by removing reads containing adapter, reads containing poly-N and low-quality reads from raw data. Paired-end clean reads were aligned to the reference genome using Hisat2 v2.0.5. The read number mapped to each gene was counted using FeatureCounts v1.5.0-p3, and then FPKM (Fragment Per Kilobase of transcript sequence per Millions base pairs sequenced) of each gene was calculated based on the length of the gene and read count mapped to this gene. The FPKM was used for estimating gene expression level. Differential expression analysis of two conditions was performed using the edgeR R package (3.18.1); Corrected P-value of 0.05 and absolute fold change of 2 were set as the threshold for significantly differential expression. Gene Ontology (GO) enrichment analysis of differentially expressed genes (DEGs) was implemented by the clusterProfiler R package.

### 2.3 Quantitative RT-PCR

RNA was isolated from cells treated or not with agonists by TRIpure reagent (Aidlab, China), and cDNA was synthesized using EasyScript Reverse Transcriptase (TransGen, China). The quantitative PCR was performed with the reagent TransStart Green qPCR SuperMix (TransGen, China) using a StepOnePlus real-time PCR System (Applied Biosystems) according to manufacturer's instructions. From the human specific, porcine specific and common DEGs, 10 genes each were selected based on the high expression levels (FPKM values), high stimulation folds by R848 and highly statistical significances so as for validation by quantitative RT-PCR. The gene transcriptional levels were calculated using ΔΔCT method.
